# Supplementary material for: The Multiple Origins of Roe Deer Populations in Western Iberia and Their Relevance for Conservation
Source: Animals (Basel). 2020 Dec 17;10(12):2419. doi: 10.3390/ani10122419 (PMC7765819; doi:10.3390/ani10122419)
Supplement: Supplementary file 1 [file animals-10-02419-s001.zip › Supplementary_Materials_Updated.docx]

**Supplementary Materials**


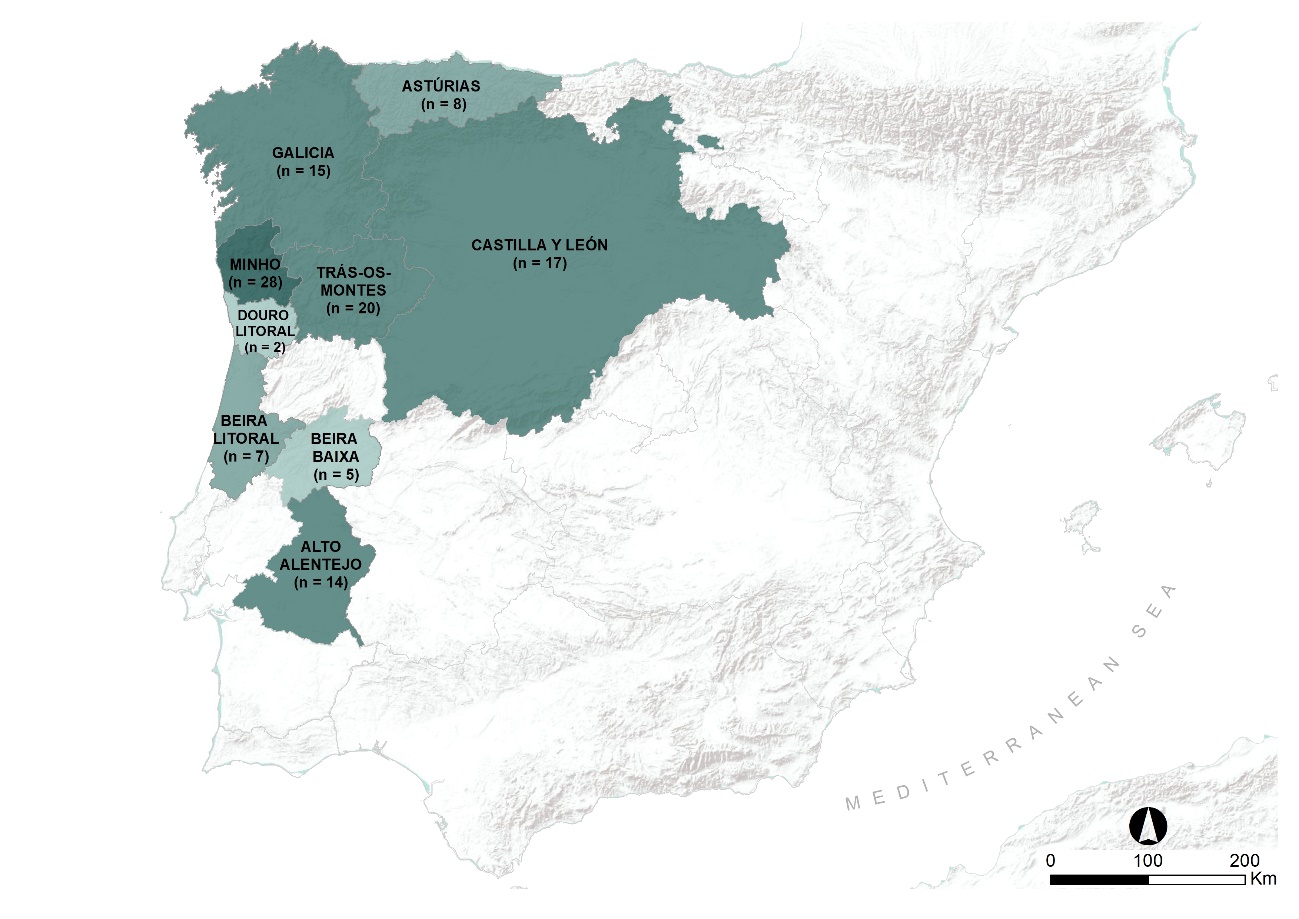


**Figure S1**. Map with number of samples of roe deer collected in each region of the Iberian Peninsula.


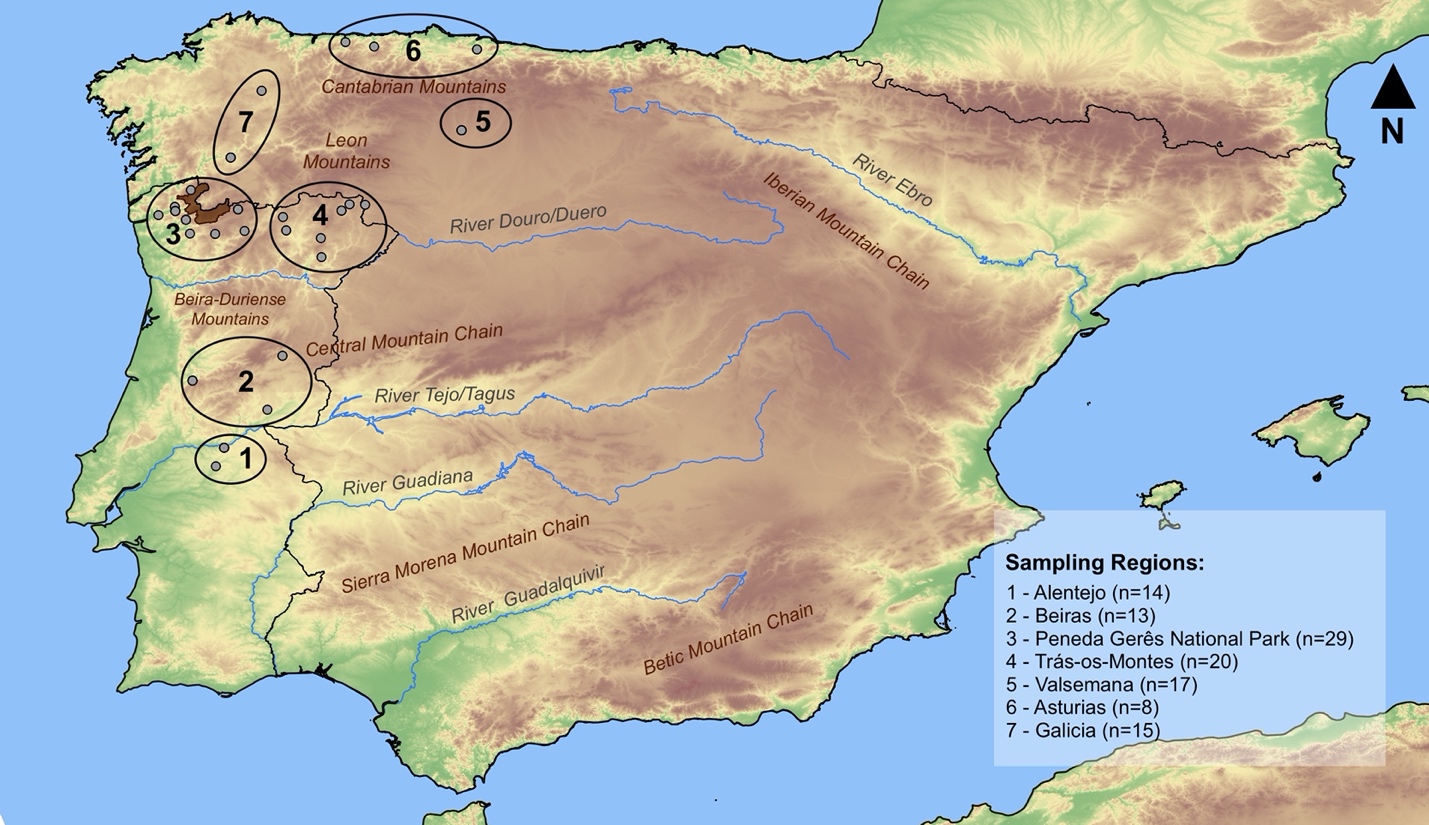


**Figure S2**. Sampling regions and locations in western Iberia, with reference to sample sizes (lower left caption) and to major rivers and mountain chains (lowlands are depicted in green; highlands are depicted in brown with higher-altitude areas, depicted in darker shades of brown). Beira-Duriense Mountains comprise, among others, the Arada, Freita and Montemuro Mountains. Peneda Gerês National Park (3) is located within the range of the Galaico-Portuguese Mountain Chain and Trás-os-Montes (4) is located within the ranges of the Galaico-Duriense Mountain Chain.

**Table S1**. Accession number for haplotypes used in this study (and haplotype designation in original study), haplotype designations used in this study, and country of origin of the haplotypes (NA – information not available).

| **Acession number** | **haplotype** | **reference** | **location** |
| --- | --- | --- | --- |
| MW160399 | H1 | this study | Portugal, Spain |
| MW160400 | H2 | this study | Portugal |
| MW160401 | H3 | this study | Portugal, Spain |
| MW160402 | H4 | this study | Portugal, Spain |
| MW160403 | H5 | this study | Portugal, Spain |
| MW160404 | H6 | this study | Portugal |
| MW160405 | H7 | this study | Portugal, Spain |
| MW160406 | H8 | this study | Portugal |
| MW160407 | H9 | this study | Portugal, Spain |
| MW160408 | H10 | this study | Portugal |
| MW160409 | H11 | this study | Portugal, Spain |
| MW160410 | H12 | this study | Portugal, Spain |
| MW160411 | H13 | this study | Portugal, Spain |
| MW160412 | H14 | this study | Portugal, Spain |
| MW160413 | H15 | this study | Portugal, Spain |
| MW160414 | H16 | this study | Portugal |
| MW160415 | H17 | this study | Portugal |
| MW160416 | H18 | this study | Portugal |
| MW160417 | H19 | this study | Portugal |
| DQ384708.1 / Sor184 | H20 | Royo et al., 2007 | Spain |
| DQ384707.1 / Sor183 | H15 | Royo et al., 2007 | Spain |
| DQ384706.1 / Sor180 | H15 | Royo et al., 2007 | Spain |
| DQ384705.1 / Sor177 | H15 | Royo et al., 2007 | Spain |
| DQ384704.1 / Sor175 | H15 | Royo et al., 2007 | Spain |
| DQ384703.1 / Sor173 | H20 | Royo et al., 2007 | Spain |
| DQ384702.1 / Sor172 | H20 | Royo et al., 2007 | Spain |
| DQ384701.1 / Sor170 | H15 | Royo et al., 2007 | Spain |
| DQ384700.1 / Sor168 | H15 | Royo et al., 2007 | Spain |
| DQ384699.1 / Sor166 | H15 | Royo et al., 2007 | Spain |
| DQ384698.1 / SeG202 | H21 | Royo et al., 2007 | Spain |
| DQ384697.1 / SeG201 | H22 | Royo et al., 2007 | Spain |
| DQ384696.1 / SeG200 | H22 | Royo et al., 2007 | Spain |
| DQ384695.1 / SeG199 | H23 | Royo et al., 2007 | Spain |
| DQ384694.1 / SeG198 | H22 | Royo et al., 2007 | Spain |
| DQ384693.1 / SeG197 | H21 | Royo et al., 2007 | Spain |
| DQ384692.1 / SeG196 | H21 | Royo et al., 2007 | Spain |
| DQ384691.1 / SeG195 | H22 | Royo et al., 2007 | Spain |
| DQ384690.1 / SeG194 | H15 | Royo et al., 2007 | Spain |
| DQ384689.1 / SeG193 | H22 | Royo et al., 2007 | Spain |
| DQ384688.1 / Lle241 | H24 | Royo et al., 2007 | Spain |
| DQ384687.1 / Lle240 | H20 | Royo et al., 2007 | Spain |
| DQ384686.1 / Lle239 | H24 | Royo et al., 2007 | Spain |
| DQ384685.1 / Lle192 | H20 | Royo et al., 2007 | Spain |
| DQ384684.1 / Lle191 | H24 | Royo et al., 2007 | Spain |
| DQ384683.1 / Lle190 | H24 | Royo et al., 2007 | Spain |
| DQ384682.1 / Lle189 | H16 | Royo et al., 2007 | Spain |
| DQ384681.1 / Lle148 | H20 | Royo et al., 2007 | Spain |
| DQ384680.1 / Lle246 | H25 | Royo et al., 2007 | Spain |
| DQ384679.1 / Lle244 | H20 | Royo et al., 2007 | Spain |
| DQ384678.1 / Ga220 | H26 | Royo et al., 2007 | Spain |
| DQ384677.1 / Ga214 | H1 | Royo et al., 2007 | Spain |
| DQ384676.1 / Ga211 | H1 | Royo et al., 2007 | Spain |
| DQ384675.1 / GA210 | H5 | Royo et al., 2007 | Spain |
| DQ384674.1 / GA209 | H15 | Royo et al., 2007 | Spain |
| DQ384673.1 / GA208 | H12 | Royo et al., 2007 | Spain |
| DQ384672.1 / GA207 | H12 | Royo et al., 2007 | Spain |
| DQ384671.1 / GA206 | H5 | Royo et al., 2007 | Spain |
| DQ384670.1 / GA205 | H27 | Royo et al., 2007 | Spain |
| DQ384669.1 / GA204 | H5 | Royo et al., 2007 | Spain |
| DQ384668.1 / CRe188 | H4 | Royo et al., 2007 | Spain |
| DQ384667.1 / CRe187 | H4 | Royo et al., 2007 | Spain |
| DQ384666.1 / CRe186 | H21 | Royo et al., 2007 | Spain |
| DQ384665.1 / CRe185 | H21 | Royo et al., 2007 | Spain |
| DQ384664.1 / Bo96 | H24 | Royo et al., 2007 | France |
| DQ384663.1 / Bo95 | H24 | Royo et al., 2007 | France |
| DQ384662.1 / Bo94 | H28 | Royo et al., 2007 | France |
| DQ384659.1 / Bo88 | H24 | Royo et al., 2007 | France |
| DQ384657.1 / Bo85 | H24 | Royo et al., 2007 | France |
| DQ384656.1 / Bo84 | H29 | Royo et al., 2007 | France |
| DQ384655.1 / Bo82 | H24 | Royo et al., 2007 | France |
| DQ384654.1 / And163 | H21 | Royo et al., 2007 | Spain |
| DQ384653.1 / And162 | H21 | Royo et al., 2007 | Spain |
| DQ384652.1 / And161 | H30 | Royo et al., 2007 | Spain |
| DQ384651.1 / And160 | H30 | Royo et al., 2007 | Spain |
| DQ384650.1 / And159 | H31 | Royo et al., 2007 | Spain |
| DQ384649.1 / And158 | H30 | Royo et al., 2007 | Spain |
| DQ384648.1 / And157 | H30 | Royo et al., 2007 | Spain |
| DQ384647.1 / And156 | H21 | Royo et al., 2007 | Spain |
| DQ384646.1 / And155 | H30 | Royo et al., 2007 | Spain |
| DQ384645.1 / And154 | H21 | Royo et al., 2007 | Spain |
| DQ384644.1 / And141 | H21 | Royo et al., 2007 | Spain |
| DQ384643.1 / And139 | H21 | Royo et al., 2007 | Spain |
| DQ384642.1 / And136 | H21 | Royo et al., 2007 | Spain |
| DQ384641.1 / And133 | H30 | Royo et al., 2007 | Spain |
| DQ384640.1 / And130 | H32 | Royo et al., 2007 | Spain |
| DQ114784.1 / Sueve69 | H5 | Royo et al., 2007 | Spain |
| DQ114783.1 / Sueve68 | H4 | Royo et al., 2007 | Spain |
| DQ114782.1 / Sueve32 | H1 | Royo et al., 2007 | Spain |
| DQ114781.1 / Sueve31 | H5 | Royo et al., 2007 | Spain |
| DQ114780.1 / Sueve30 | H33 | Royo et al., 2007 | Spain |
| DQ114779.1 / Sueve25 | H5 | Royo et al., 2007 | Spain |
| DQ114778.1 / Sueve152 | H5 | Royo et al., 2007 | Spain |
| DQ114777.1 / Sueve151 | H4 | Royo et al., 2007 | Spain |
| DQ114776.1 / Sueve150 | H1 | Royo et al., 2007 | Spain |
| DQ114775.1 / Sueve129 | H5 | Royo et al., 2007 | Spain |
| DQ114774.1 / Valdes9 | H12 | Royo et al., 2007 | Spain |
| DQ114773.1 / Valdes8 | H3 | Royo et al., 2007 | Spain |
| DQ114772.1 / Valdes7 | H12 | Royo et al., 2007 | Spain |
| DQ114771.1 / Valdes6 | H12 | Royo et al., 2007 | Spain |
| DQ114770.1 / Valdes5 | H3 | Royo et al., 2007 | Spain |
| DQ114769.1 / Valdes4 | H12 | Royo et al., 2007 | Spain |
| DQ114768.1 / Valdes2 | H12 | Royo et al., 2007 | Spain |
| DQ114767.1 / Valdes11 | H12 | Royo et al., 2007 | Spain |
| DQ114766.1 / Valdes10 | H3 | Royo et al., 2007 | Spain |
| DQ114765.1 / Valdes1 | H12 | Royo et al., 2007 | Spain |
| DQ114764.1 / Picos / de / Europa / 81 | H4 | Royo et al., 2007 | Spain |
| DQ114763.1 / Picos / de / Europa / 80 | H34 | Royo et al., 2007 | Spain |
| DQ114762.1 / Picos / de / Europa / 79 | H5 | Royo et al., 2007 | Spain |
| DQ114761.1 / Picos / de / Europa / 74 | H1 | Royo et al., 2007 | Spain |
| DQ114760.1 / Picos / de / Europa / 46 | H13 | Royo et al., 2007 | Spain |
| DQ114759.1 / Picos / de / Europa / 45 | H13 | Royo et al., 2007 | Spain |
| DQ114758.1 / Picos / de / Europa / 44 | H13 | Royo et al., 2007 | Spain |
| DQ114757.1 / Picos / de / Europa / 43 | H5 | Royo et al., 2007 | Spain |
| DQ114756.1 / Picos / de / Europa / 42 | H13 | Royo et al., 2007 | Spain |
| DQ114755.1 / Picos / de / Europa / 39 | H20 | Royo et al., 2007 | Spain |
| DQ114754.1 / Muniellos127 | H20 | Royo et al., 2007 | Spain |
| DQ114753.1 / Muniellos124 | H15 | Royo et al., 2007 | Spain |
| DQ114752.1 / Muniellos119 | H3 | Royo et al., 2007 | Spain |
| DQ114751.1 / Muniellos118 | H3 | Royo et al., 2007 | Spain |
| DQ114750.1 / Muniellos113 | H1 | Royo et al., 2007 | Spain |
| DQ114749.1 / Muniellos110 | H15 | Royo et al., 2007 | Spain |
| DQ114748.1 / Muniellos105 | H1 | Royo et al., 2007 | Spain |
| DQ114747.1 / Muniellos104 | H12 | Royo et al., 2007 | Spain |
| DQ114746.1 / Muniellos101 | H12 | Royo et al., 2007 | Spain |
| DQ114745.1 / Muniellos100 | H3 | Royo et al., 2007 | Spain |
| Z70318.1 | H35 | Douzery and Randi 1997 | NA |
| AY625892.1 / H161 | H36 | Randi et al., 2004 | NA |
| AY625891.1 / H160 | H30 | Randi et al., 2004 | NA |
| AY625890.1 / H159 | H22 | Randi et al., 2004 | Spain |
| AY625889.1 / H158 | H20 | Randi et al., 2004 | Spain |
| AY625888.1 / H157 | H37 | Randi et al., 2004 | NA |
| AY625887.1 / H156 | H38 | Randi et al., 2004 | NA |
| AY625886.1 / H155 | H39 | Randi et al., 2004 | NA |
| AY625885.1 / H154 | H40 | Randi et al., 2004 | NA |
| AY625884.1 / H153 | H41 | Randi et al., 2004 | NA |
| AY625883.1 / H152 | H42 | Randi et al., 2004 | NA |
| AY625882.1 / H151 | H43 | Randi et al., 2004 | NA |
| AY625881.1 / H150 | H41 | Randi et al., 2004 | NA |
| AY625880.1 / H149 | H38 | Randi et al., 2004 | NA |
| AY625879.1 / H148 | H20 | Randi et al., 2004 | NA |
| AY625878.1 / H147 | H38 | Randi et al., 2004 | NA |
| AY625877.1 / H146 | H39 | Randi et al., 2004 | NA |
| AY625876.1 / H145 | H44 | Randi et al., 2004 | Serbia |
| AY625875.1 / H144 | H45 | Randi et al., 2004 | NA |
| AY625874.1 / H143 | H46 | Randi et al., 2004 | NA |
| AY625873.1 / H142 | H47 | Randi et al., 2004 | NA |
| AY625872.1 / H141 | H44 | Randi et al., 2004 | Serbia |
| AY625871.1 / H140 | H41 | Randi et al., 2004 | NA |
| AY625870.1 / H139 | H48 | Randi et al., 2004 | NA |
| AY625869.1 / H138 | H49 | Randi et al., 2004 | Serbia |
| AY625868.1 / H137 | H20 | Randi et al., 2004 | Serbia |
| AY625867.1 / H136 | H29 | Randi et al., 2004 | NA |
| AY625866.1 / H135 | H50 | Randi et al., 2004 | NA |
| AY625865.1 / H134 | H44 | Randi et al., 2004 | NA |
| AY625864.1 / H133 | H49 | Randi et al., 2004 | NA |
| AY625863.1 / H132 | H41 | Randi et al., 2004 | Serbia |
| AY625862.1 / H131 | H41 | Randi et al., 2004 | Serbia |
| AY625861.1 / H130 | H51 | Randi et al., 2004 | NA |
| AY625860.1 / H129 | H52 | Randi et al., 2004 | NA |
| AY625859.1 / H128 | H47 | Randi et al., 2004 | NA |
| AY625858.1 / H127 | H38 | Randi et al., 2004 | NA |
| AY625857.1 / H126 | H53 | Randi et al., 2004 | NA |
| AY625856.1 / H125 | H54 | Randi et al., 2004 | NA |
| AY625855.1 / H124 | H44 | Randi et al., 2004 | NA |
| AY625854.1 / H123 | H35 | Randi et al., 2004 | NA |
| AY625853.1 / H122 | H35 | Randi et al., 2004 | Serbia |
| AY625852.1 / H121 | H29 | Randi et al., 2004 | Serbia |
| AY625851.1 / H120 | H38 | Randi et al., 2004 | Serbia |
| AY625850.1 / H119 | H45 | Randi et al., 2004 | Serbia |
| AY625849.1 / H118 | H55 | Randi et al., 2004 | Serbia |
| AY625848.1 / H117 | H49 | Randi et al., 2004 | Serbia |
| AY625847.1 / H116 | H39 | Randi et al., 2004 | Serbia |
| AY625846.1 / H115 | H48 | Randi et al., 2004 | Serbia |
| AY625845.1 / H114 | H56 | Randi et al., 2004 | NA |
| AY625844.1 / H113 | H38 | Randi et al., 2004 | NA |
| AY625843.1 / H112 | H39 | Randi et al., 2004 | NA |
| AY625842.1 / H111 | H57 | Randi et al., 2004 | Serbia |
| AY625841.1 / H110 | H38 | Randi et al., 2004 | NA |
| AY625840.1 / H109 | H48 | Randi et al., 2004 | Serbia |
| AY625839.1 / H108 | H20 | Randi et al., 2004 | NA |
| AY625838.1 / H107 | H49 | Randi et al., 2004 | Serbia |
| AY625837.1 / H106 | H40 | Randi et al., 2004 | NA |
| AY625836.1 / H105 | H35 | Randi et al., 2004 | NA |
| AY625835.1 / H104 | H54 | Randi et al., 2004 | Serbia |
| AY625834.1 / H103 | H40 | Randi et al., 2004 | Serbia |
| AY625833.1 / H102 | H38 | Randi et al., 2004 | Serbia |
| AY625832.1 / H101 | H49 | Randi et al., 2004 | NA |
| AY625831.1 / H100 | H10 | Randi et al., 2004 | NA |
| AY625830.1 / H99 | H58 | Randi et al., 2004 | NA |
| AY625829.1 / H98 | H59 | Randi et al., 2004 | NA |
| AY625828.1 / H97 | H59 | Randi et al., 2004 | France |
| AY625827.1 / H96 | H24 | Randi et al., 2004 | NA |
| AY625826.1 / H95 | H2 | Randi et al., 2004 | France |
| AY625825.1 / H94 | H60 | Randi et al., 2004 | NA |
| AY625824.1 / H93 | H4 | Randi et al., 2004 | NA |
| AY625823.1 / H92 | H4 | Randi et al., 2004 | Spain |
| AY625822.1 / H91 | H61 | Randi et al., 2004 | NA |
| AY625821.1 / H90 | H48 | Randi et al., 2004 | Serbia |
| AY625820.1 / H89 | H40 | Randi et al., 2004 | Serbia |
| AY625819.1 / H88 | H62 | Randi et al., 2004 | NA |
| AY625818.1 / H87 | H35 | Randi et al., 2004 | Serbia |
| AY625817.1 / H86 | H53 | Randi et al., 2004 | Serbia |
| AY625816.1 / H85 | H35 | Randi et al., 2004 | Serbia |
| AY625815.1 / H84 | H63 | Randi et al., 2004 | Serbia |
| AY625814.1 / H83 | H40 | Randi et al., 2004 | Serbia |
| AY625813.1 / H82 | H47 | Randi et al., 2004 | Serbia |
| AY625812.1 / H81 | H45 | Randi et al., 2004 | NA |
| AY625811.1 / H80 | H49 | Randi et al., 2004 | Serbia |
| AY625810.1 / H79 | H64 | Randi et al., 2004 | NA |
| AY625809.1 / H78 | H7 | Randi et al., 2004 | Iberia |
| AY625808.1 / H77 | H65 | Randi et al., 2004 | NA |
| AY625807.1 / H76 | H3 | Randi et al., 2004 | NA |
| AY625806.1 / H75 | H8 | Randi et al., 2004 | NA |
| AY625805.1 / H74 | H15 | Randi et al., 2004 | Spain |
| AY625804.1 / H73 | H66 | Randi et al., 2004 | NA |
| AY625803.1 / H72 | H67 | Randi et al., 2004 | NA |
| AY625802.1 / H71 | H11 | Randi et al., 2004 | Spain/Portugal |
| AY625801.1 / H70 | H8 | Randi et al., 2004 | Portugal |
| AY625800.1 / H69 | H8 | Randi et al., 2004 | Portugal |
| AY625799.1 / H68 | H68 | Randi et al., 2004 | NA |
| AY625798.1 / H67 | H69 | Randi et al., 2004 | Italy |
| AY625797.1 / H66 | H20 | Randi et al., 2004 | NA |
| AY625796.1 / H65 | H49 | Randi et al., 2004 | NA |
| AY625795.1 / H64 | H70 | Randi et al., 2004 | NA |
| AY625794.1 / H63 | H71 | Randi et al., 2004 | Denmark |
| AY625793.1 / H62 | H72 | Randi et al., 2004 | Denmark |
| AY625792.1 / H61 | H73 | Randi et al., 2004 | NA |
| AY625791.1 / H60 | H74 | Randi et al., 2004 | Italy |
| AY625790.1 / H59 | H75 | Randi et al., 2004 | NA |
| AY625789.1 / H58 | H76 | Randi et al., 2004 | NA |
| AY625788.1 / H57 | H77 | Randi et al., 2004 | Italy |
| AY625787.1 / H56 | H78 | Randi et al., 2004 | NA |
| AY625786.1 / H55 | H10 | Randi et al., 2004 | NA |
| AY625785.1 / H54 | H49 | Randi et al., 2004 | Germany/Italy/Serbia |
| AY625784.1 / H53 | H29 | Randi et al., 2004 | NA |
| AY625783.1 / H52 | H24 | Randi et al., 2004 | NA |
| AY625782.1 / H51 | H20 | Randi et al., 2004 | NA |
| AY625781.1 / H50 | H79 | Randi et al., 2004 | NA |
| AY625780.1 / H49 | H29 | Randi et al., 2004 | NA |
| AY625779.1 / H48 | H49 | Randi et al., 2004 | Germany/Italy/Serbia |
| AY625778.1 / H47 | H20 | Randi et al., 2004 | Denmark/Serbia |
| AY625777.1 / H46 | H24 | Randi et al., 2004 | NA |
| AY625776.1 / H45 | H74 | Randi et al., 2004 | Italy |
| AY625775.1 / H44 | H80 | Randi et al., 2004 | NA |
| AY625774.1 / H43 | H81 | Randi et al., 2004 | Italy |
| AY625773.1 / H42 | H80 | Randi et al., 2004 | Italy |
| AY625772.1 / H41 | H24 | Randi et al., 2004 | Germany/Italy |
| AY625771.1 / H40 | H82 | Randi et al., 2004 | Italy |
| AY625770.1 / H39 | H83 | Randi et al., 2004 | NA |
| AY625769.1 / H38 | H49 | Randi et al., 2004 | Sweden |
| AY625768.1 / H37 | H35 | Randi et al., 2004 | Italy/Serbia |
| AY625767.1 / H36 | H84 | Randi et al., 2004 | NA |
| AY625766.1 / H35 | H80 | Randi et al., 2004 | Italy |
| AY625765.1 / H34 | H85 | Randi et al., 2004 | NA |
| AY625764.1 / H33 | H86 | Randi et al., 2004 | NA |
| AY625763.1 / H32 | H87 | Randi et al., 2004 | NA |
| AY625762.1 / H31 | H67 | Randi et al., 2004 | Italy |
| AY625761.1 / H30 | H16 | Randi et al., 2004 | Italy/Serbia |
| AY625760.1 / H29 | H51 | Randi et al., 2004 | Italy/Serbia |
| AY625759.1 / H28 | H39 | Randi et al., 2004 | Italy/Serbia |
| AY625758.1 / H27 | H57 | Randi et al., 2004 | Italy/Serbia |
| AY625757.1 / H26 | H20 | Randi et al., 2004 | NA |
| AY625756.1 / H25 | H88 | Randi et al., 2004 | Italy |
| AY625755.1 / H24 | H89 | Randi et al., 2004 | Germany/Italy |
| AY625754.1 / H23 | H29 | Randi et al., 2004 | Italy/Serbia |
| AY625753.1 / H22 | H90 | Randi et al., 2004 | France |
| AY625752.1 / H21 | H20 | Randi et al., 2004 | France/Germany/Serbia |
| AY625751.1 / H20 | H6 | Randi et al., 2004 | NA |
| AY625750.1 / H19 | H20 | Randi et al., 2004 | France |
| AY625749.1 / H18 | H24 | Randi et al., 2004 | Italy |
| AY625748.1 / H17 | H42 | Randi et al., 2004 | Italy |
| AY625747.1 / H16 | H29 | Randi et al., 2004 | Germany/Italy |
| AY625746.1 / H15 | H80 | Randi et al., 2004 | Italy |
| AY625745.1 / H14 | H35 | Randi et al., 2004 | Italy |
| AY625744.1 / H13 | H67 | Randi et al., 2004 | Italy |
| AY625743.1 / H12 | H91 | Randi et al., 2004 | Italy |
| AY625742.1 / H11 | H92 | Randi et al., 2004 | Greece |
| AY625741.1 / H10 | H93 | Randi et al., 2004 | Greece |
| AY625740.1 / H9 | H94 | Randi et al., 2004 | NA |
| AY625739.1 / H8 | H95 | Randi et al., 2004 | NA |
| AY625738.1 / H7 | H96 | Randi et al., 2004 | Denmark/Germany/Spain |
| AY625737.1 / H6 | H3 | Randi et al., 2004 | Spain/Portugal |
| AY625736.1 / H5 | H5 | Randi et al., 2004 | Spain |
| AY625735.1 / H4 | H1 | Randi et al., 2004 | Spain |
| AY625734.1 / H3 | H1 | Randi et al., 2004 | Spain/Portugal |
| AY625733.1 / H2 | H5 | Randi et al., 2004 | Spain/Portugal |
| AY625732.1 / H1 | H12 | Randi et al., 2004 | Spain |
| JX971589.1 / h1 | H20 | Baker & Hoelzel 2013 | Britain |
| JX971590.1 / h2 | H24 | Baker & Hoelzel 2013 | Britain |
| JX971591.1 / h3 | H89 | Baker & Hoelzel 2013 | Britain |
| JX971592.1 / h4 | H24 | Baker & Hoelzel 2013 | Britain |
| JX971593.1 / h5 | H97 | Baker & Hoelzel 2013 | Britain |
| JX971594.1 / h6 | H40 | Baker & Hoelzel 2013 | Britain |
| JX971595.1 / h7 | H20 | Baker & Hoelzel 2013 | Britain |
| JX971596.1 / h8 | H40 | Baker & Hoelzel 2013 | Britain |
| JX971597.1 / h9 | H97 | Baker & Hoelzel 2013 | Britain |
| JX971598.1 / h10 | H49 | Baker & Hoelzel 2013 | Britain |
| JX971599.1 / h11 | H20 | Baker & Hoelzel 2013 | Britain |
| JX971600.1 / h12 | H98 | Baker & Hoelzel 2013 | Britain |
| JX971601.1 / h13 | H49 | Baker & Hoelzel 2013 | Britain |
| JX971603.1 / h15 | H99 | Baker & Hoelzel 2013 | Britain |
| JX971604.1 / h16 | H20 | Baker & Hoelzel 2013 | Britain |
| JX971605.1 / h17 | H50 | Baker & Hoelzel 2013 | Britain |
| JX971606.1 / h18 | H100 | Baker & Hoelzel 2013 | Britain |
| JX971607.1 / h19 | H40 | Baker & Hoelzel 2013 | Britain |
| JX971608.1 / h20 | H20 | Baker & Hoelzel 2013 | Britain |
| JX971609.1 / h21 | H20 | Baker & Hoelzel 2013 | Britain |
| JX971610.1 / h22 | H101 | Baker & Hoelzel 2013 | Britain |
| JX971611.1 / h23 | H102 | Baker & Hoelzel 2013 | Britain |
| JX971612.1 / h24 | H97 | Baker & Hoelzel 2013 | Britain |
| JX971613.1 / h25 | H102 | Baker & Hoelzel 2013 | Britain |
| JX971614.1 / h26 | H97 | Baker & Hoelzel 2013 | Britain |
| JX971615.1 / h27 | H20 | Baker & Hoelzel 2013 | Britain |
| KC178710.1 / H162 | H103 | Mucci et al., 2012 | Italy |
| KC178711.1 / H163 | H24 | Mucci et al., 2012 | Italy |
| KC178712.1 / H164 | H104 | Mucci et al., 2012 | Italy |
| KC178713.1 / H165 | H105 | Mucci et al., 2012 | Italy |
| KC178714.1 / H166 | H106 | Mucci et al., 2012 | Italy |
| KF961012.1 / a1 | H49 | Baker & Hoelzel 2014 | Britain |
| KF961013.1 / a2 | H20 | Baker & Hoelzel 2014 | Britain |
| KF961014.1 / a3 | H24 | Baker & Hoelzel 2014 | Britain |
| KF961015.1 / a4 | H107 | Baker & Hoelzel 2014 | Britain |
| KF961016.1 / a5 | H63 | Baker & Hoelzel 2014 | Britain |
| KF961017.1 / a6 | H108 | Baker & Hoelzel 2014 | Britain |
| KF961018.1 / a7 | H109 | Baker & Hoelzel 2014 | Britain |
| KF961019.1 / a8 | H110 | Baker & Hoelzel 2014 | Britain |
| KF961020.1 / a9 | H111 | Baker & Hoelzel 2014 | Britain |
| KF961021.1 / a10 | H112 | Baker & Hoelzel 2014 | Britain |
| KF961022.1 / a11 | H113 | Baker & Hoelzel 2014 | Britain |
| KF961023.1 / a12 | H114 | Baker & Hoelzel 2014 | Britain |
| KF961024.1 / a13 | H40 | Baker & Hoelzel 2014 | Britain |
| KF961025.1 / a14 | H115 | Baker & Hoelzel 2014 | Britain |
| KF961026.1 / a15 | H116 | Baker & Hoelzel 2014 | Britain |
| KF961027.1 / a16 | H97 | Baker & Hoelzel 2014 | Britain |
| KF961028.1 / a17 | H50 | Baker & Hoelzel 2014 | Britain |
| KF961029.1 / a18 | H117 | Baker & Hoelzel 2014 | Britain |
| KF961030.1 / a19 | H118 | Baker & Hoelzel 2014 | Britain |
| KF961031.1 / a20 | H119 | Baker & Hoelzel 2014 | Britain |
| KF961032.1 / a21 | H120 | Baker & Hoelzel 2014 | Britain |
| KF961033.1 / a22 | H121 | Baker & Hoelzel 2014 | Britain |
| KF961034.1 / a23 | H122 | Baker & Hoelzel 2014 | Britain |
| KF961035.1 / a24 | H123 | Baker & Hoelzel 2014 | Britain |
| KM873736.1 / B01 | H124 | Biosa et al., 2015 | Italy |
| KM873737.1 / B02 | H125 | Biosa et al., 2015 | Italy |
| KM873738.1 / B03 | H126 | Biosa et al., 2015 | Italy |
| KM873739.1 / B04 | H127 | Biosa et al., 2015 | Italy |
| KM873740.1 / B05 | H128 | Biosa et al., 2015 | Italy |
| KM873741.1 / B06 | H24 | Biosa et al., 2015 | Italy |
| KM873742.1 / B07 | H125 | Biosa et al., 2015 | Italy |
| KM873743.1 / B08 | H129 | Biosa et al., 2015 | Italy |
| KM873744.1 / B09 | H130 | Biosa et al., 2015 | Italy |
| KM873745.1 / B10 | H24 | Biosa et al., 2015 | Italy |
| KM873746.1 / B11 | H131 | Biosa et al., 2015 | Italy |
| KM873747.1 / B12 | H132 | Biosa et al., 2015 | Italy |
| HM121224.1 / 2208CAL | H35 | Vernesi et al., 2016 | Italy |
| HM121225.1 / 2207CAL | H35 | Vernesi et al., 2016 | Italy |
| HM121226.1 / 2206CAL | H35 | Vernesi et al., 2016 | Italy |
| HM121227.1 / 2205CAL | H35 | Vernesi et al., 2016 | Italy |
| HM121228.1 / 2204CAL | H35 | Vernesi et al., 2016 | Italy |
| HM121229.1 / 2203CAL | H35 | Vernesi et al., 2016 | Italy |
| HM121230.1 / 2202CAL | H35 | Vernesi et al., 2016 | Italy |
| HM121231.1 / 2201CAL | H35 | Vernesi et al., 2016 | Italy |
| HM121232.1 / 2200CAL | H35 | Vernesi et al., 2016 | Italy |
| HM121233.1 / 2025CAL | H35 | Vernesi et al., 2016 | Italy |
| HM121234.1 / 2024CAL | H35 | Vernesi et al., 2016 | Italy |
| HM121235.1 / 2020CAL | H35 | Vernesi et al., 2016 | Italy |
| HM121236.1 / 2019CAL | H35 | Vernesi et al., 2016 | Italy |
| HM121237.1 / 2014CAL | H35 | Vernesi et al., 2016 | Italy |
| HM121238.1 / 2009CAL | H35 | Vernesi et al., 2016 | Italy |
| HM121239.1 / 2004CAL | H35 | Vernesi et al., 2016 | Italy |
| HM121240.1 / 2127CAL | H35 | Vernesi et al., 2016 | Italy |
| HM121241.1 / 2107CAL | H35 | Vernesi et al., 2016 | Italy |
| HM121242.1 / 2199CAL | H35 | Vernesi et al., 2016 | Italy |
| HM121243.1 / 2039CAL | H35 | Vernesi et al., 2016 | Italy |
| HM121244.1 / 2034CAL | H35 | Vernesi et al., 2016 | Italy |
| HM121245.1 / 2037CAL | H35 | Vernesi et al., 2016 | Italy |
| HM121246.1 / 2031CAL | H35 | Vernesi et al., 2016 | Italy |
| HM121247.1 / 2032CAL | H35 | Vernesi et al., 2016 | Italy |
| HM121248.1 / 2237CAL | H35 | Vernesi et al., 2016 | Italy |
| HM121249.1 / 2028CAL | H35 | Vernesi et al., 2016 | Italy |
| HM121250.1 / 2027CAL | H35 | Vernesi et al., 2016 | Italy |
| HM121251.1 / 2038CAL | H67 | Vernesi et al., 2016 | Italy |
| HM121252.1 / 2210PRI | H35 | Vernesi et al., 2016 | Italy |
| HM121253.1 / 2218PRI | H35 | Vernesi et al., 2016 | Italy |
| HM121254.1 / 2217PRI | H35 | Vernesi et al., 2016 | Italy |
| HM121255.1 / 2215PRI | H35 | Vernesi et al., 2016 | Italy |
| HM121256.1 / 2213PRI | H35 | Vernesi et al., 2016 | Italy |
| HM121257.1 / 2212PRI | H35 | Vernesi et al., 2016 | Italy |
| HM121258.1 / 2240PRI | H35 | Vernesi et al., 2016 | Italy |
| HM121259.1 / 2222PRI | H35 | Vernesi et al., 2016 | Italy |
| HM121260.1 / 2238PRI | H35 | Vernesi et al., 2016 | Italy |
| HM121261.1 / 2221PRI | H67 | Vernesi et al., 2016 | Italy |
| HM121262.1 / 2219PRI | H49 | Vernesi et al., 2016 | Italy |
| HM121263.1 / 2241PRI | H49 | Vernesi et al., 2016 | Italy |
| HM121264.1 / 2137PRI | H133 | Vernesi et al., 2016 | Italy |
| HM121265.1 / 2209PRI | H24 | Vernesi et al., 2016 | Italy |
| HM121266.1 / 2078TNO | H35 | Vernesi et al., 2016 | Italy |
| HM121267.1 / 2081TNO | H35 | Vernesi et al., 2016 | Italy |
| HM121268.1 / 2076TNO | H35 | Vernesi et al., 2016 | Italy |
| HM121269.1 / 2073TNO | H35 | Vernesi et al., 2016 | Italy |
| HM121270.1 / 2070TNO | H35 | Vernesi et al., 2016 | Italy |
| HM121271.1 / 2088TNO | H35 | Vernesi et al., 2016 | Italy |
| HM121272.1 / 2065TNO | H35 | Vernesi et al., 2016 | Italy |
| HM121273.1 / 2063TNO | H35 | Vernesi et al., 2016 | Italy |
| HM121274.1 / 2062TNO | H35 | Vernesi et al., 2016 | Italy |
| HM121275.1 / 2060TNO | H35 | Vernesi et al., 2016 | Italy |
| HM121276.1 / 2057TNO | H35 | Vernesi et al., 2016 | Italy |
| HM121277.1 / 2056TNO | H35 | Vernesi et al., 2016 | Italy |
| HM121278.1 / 2055TNO | H35 | Vernesi et al., 2016 | Italy |
| HM121279.1 / 2054TNO | H35 | Vernesi et al., 2016 | Italy |
| HM121280.1 / 2052TNO | H35 | Vernesi et al., 2016 | Italy |
| HM121281.1 / 2051TNO | H35 | Vernesi et al., 2016 | Italy |
| HM121282.1 / 2045TNO | H35 | Vernesi et al., 2016 | Italy |
| HM121283.1 / 2047TNO | H35 | Vernesi et al., 2016 | Italy |
| HM121284.1 / 2041TNO | H35 | Vernesi et al., 2016 | Italy |
| HM121285.1 / 2042TNO | H89 | Vernesi et al., 2016 | Italy |
| HM121286.1 / 2080TNO | H89 | Vernesi et al., 2016 | Italy |
| HM121287.1 / 2084TNO | H89 | Vernesi et al., 2016 | Italy |
| HM121288.1 / 2044TNO | H29 | Vernesi et al., 2016 | Italy |
| HM121289.1 / 2049TNO | H29 | Vernesi et al., 2016 | Italy |
| HM121290.1 / 2072TNO | H29 | Vernesi et al., 2016 | Italy |
| HM121291.1 / 2079TNO | H29 | Vernesi et al., 2016 | Italy |
| HM121292.1 / 2083TNO | H29 | Vernesi et al., 2016 | Italy |
| HM121293.1 / 2086TNO | H29 | Vernesi et al., 2016 | Italy |
| HM121294.1 / 2223TNO | H29 | Vernesi et al., 2016 | Italy |
| HM121295.1 / 2095TNO | H134 | Vernesi et al., 2016 | Italy |
| HM121296.1 / 2067TNO | H67 | Vernesi et al., 2016 | Italy |
| HM121297.1 / 2134VFF | H35 | Vernesi et al., 2016 | Italy |
| HM121298.1 / 2220VFF | H35 | Vernesi et al., 2016 | Italy |
| HM121299.1 / 2214VFF | H35 | Vernesi et al., 2016 | Italy |
| HM121300.1 / 2242VFF | H35 | Vernesi et al., 2016 | Italy |
| HM121301.1 / 2243VFF | H35 | Vernesi et al., 2016 | Italy |
| HM121302.1 / 2226VFF | H67 | Vernesi et al., 2016 | Italy |
| HM121303.1 / 2227VFF | H67 | Vernesi et al., 2016 | Italy |
| HM121304.1 / 2211VFF | H49 | Vernesi et al., 2016 | Italy |
| HM121305.1 / 2224VFF | H49 | Vernesi et al., 2016 | Italy |
| HM121306.1 / 2225VFF | H49 | Vernesi et al., 2016 | Italy |
| HM121307.1 / 2234VFF | H49 | Vernesi et al., 2016 | Italy |
| HM121308.1 / 2141VFF | H49 | Vernesi et al., 2016 | Italy |
| HM121309.1 / 2129VFF | H35 | Vernesi et al., 2016 | Italy |
| HM121310.1 / 2132VFF | H133 | Vernesi et al., 2016 | Italy |
| HM121311.1 / 2131VFF | H24 | Vernesi et al., 2016 | Italy |
| HM121312.1 / 2174VS | H35 | Vernesi et al., 2016 | Italy |
| HM121313.1 / 2244VS | H35 | Vernesi et al., 2016 | Italy |
| HM121314.1 / 2229VS | H67 | Vernesi et al., 2016 | Italy |
| HM121315.1 / 2140VS | H67 | Vernesi et al., 2016 | Italy |
| HM121316.1 / 2183VS | H67 | Vernesi et al., 2016 | Italy |
| HM121317.1 / 2146VS | H67 | Vernesi et al., 2016 | Italy |
| HM121318.1 / 2145VS | H67 | Vernesi et al., 2016 | Italy |
| HM121319.1 / 2235VS | H67 | Vernesi et al., 2016 | Italy |
| HM121320.1 / 2135VS | H135 | Vernesi et al., 2016 | Italy |
| HM121321.1 / 2188VS | H136 | Vernesi et al., 2016 | Italy |
| HM121322.1 / 2187VS | H89 | Vernesi et al., 2016 | Italy |
| HM121323.1 / 2133VS | H89 | Vernesi et al., 2016 | Italy |
| HM121324.1 / 2152VS | H89 | Vernesi et al., 2016 | Italy |
| HM121325.1 / 2144VS | H49 | Vernesi et al., 2016 | Italy |
| HM121326.1 / 2194VS | H49 | Vernesi et al., 2016 | Italy |
| HM121327.1 / 2195VS | H137 | Vernesi et al., 2016 | Italy |
| HM121328.1 / 2176VS | H29 | Vernesi et al., 2016 | Italy |
| HM121329.1 / 2179VS | H29 | Vernesi et al., 2016 | Italy |
| HM121330.1 / 2230VS | H29 | Vernesi et al., 2016 | Italy |
| HM121331.1 / 2231VS | H29 | Vernesi et al., 2016 | Italy |
| HM121332.1 / 2232VS | H29 | Vernesi et al., 2016 | Italy |
| HM121333.1 / 2233VS | H29 | Vernesi et al., 2016 | Italy |
| HM121334.1 / 2236VS | H29 | Vernesi et al., 2016 | Italy |
| HM121335.1 / 2189VS | H29 | Vernesi et al., 2016 | Italy |
| HM121336.1 / 2190VS | H29 | Vernesi et al., 2016 | Italy |
| HM121337.1 / 2191VS | H29 | Vernesi et al., 2016 | Italy |
| HM121338.1 / 2228VS | H138 | Vernesi et al., 2016 | Italy |
| HM121339.1 / 2156VS | H139 | Vernesi et al., 2016 | Italy |
| HM121340.1 / 2178VS | H78 | Vernesi et al., 2016 | Italy |
| MH460559.1 / AP01 | H140 | Tsaparis et al., 2019 | Greece |
| MH460560.1 / L01 | H141 | Tsaparis et al., 2019 | Greece |
| MH460561.1 / L02 | H141 | Tsaparis et al., 2019 | Greece |
| MH460562.1 / L03 | H141 | Tsaparis et al., 2019 | Greece |
| MH460563.1 / L04 | H42 | Tsaparis et al., 2019 | Greece |
| MH460564.1 / L05 | H141 | Tsaparis et al., 2019 | Greece |
| MH460565.1 / L06 | H141 | Tsaparis et al., 2019 | Greece |
| MH460566.1 / L07 | H141 | Tsaparis et al., 2019 | Greece |
| MH460567.1 / L08 | H42 | Tsaparis et al., 2019 | Greece |
| MH460568.1 / L09 | H42 | Tsaparis et al., 2019 | Greece |
| MH460569.1 / L10 | H141 | Tsaparis et al., 2019 | Greece |
| MH460570.1 / L11 | H141 | Tsaparis et al., 2019 | Greece |
| MH460571.1 / L12 | H142 | Tsaparis et al., 2019 | Greece |
| MH460572.1 / L15 | H48 | Tsaparis et al., 2019 | Greece |
| MH460573.1 / L17 | H141 | Tsaparis et al., 2019 | Greece |
| MH460574.1 / L18 | H141 | Tsaparis et al., 2019 | Greece |
| MH460575.1 / L19 | H141 | Tsaparis et al., 2019 | Greece |
| MH460576.1 / L20 | H51 | Tsaparis et al., 2019 | Greece |
| MH460577.1 / L21 | H143 | Tsaparis et al., 2019 | Greece |
| MH460578.1 / L22 | H141 | Tsaparis et al., 2019 | Greece |
| MH460579.1 / E01 | H144 | Tsaparis et al., 2019 | Greece |
| MH460580.1 / K01 | H145 | Tsaparis et al., 2019 | Greece |
| MH460581.1 / K02 | H145 | Tsaparis et al., 2019 | Greece |
| MH460582.1 / K03 | H143 | Tsaparis et al., 2019 | Greece |
| MH460583.1 / O01 | H42 | Tsaparis et al., 2019 | Greece |
| MH460584.1 / O02 | H57 | Tsaparis et al., 2019 | Greece |
| MH460585.1 / O03 | H42 | Tsaparis et al., 2019 | Greece |
| MH460586.1 / O05 | H42 | Tsaparis et al., 2019 | Greece |
| MH460587.1 / O06 | H42 | Tsaparis et al., 2019 | Greece |
| MH460588.1 / O07 | H42 | Tsaparis et al., 2019 | Greece |
| MH460589.1 / O08 | H57 | Tsaparis et al., 2019 | Greece |
| MH460590.1 / O09 | H57 | Tsaparis et al., 2019 | Greece |
| MH460591.1 / O10 | H42 | Tsaparis et al., 2019 | Greece |
| MH460592.1 / O11 | H42 | Tsaparis et al., 2019 | Greece |
| MH460593.1 / O12 | H57 | Tsaparis et al., 2019 | Greece |
| MH460594.1 / O13 | H57 | Tsaparis et al., 2019 | Greece |
| MH460595.1 / O14 | H42 | Tsaparis et al., 2019 | Greece |
| MH460596.1 / O15 | H57 | Tsaparis et al., 2019 | Greece |
| MH460597.1 / O16 | H42 | Tsaparis et al., 2019 | Greece |
| MH460598.1 / O17 | H57 | Tsaparis et al., 2019 | Greece |
| MH460599.1 / O18 | H42 | Tsaparis et al., 2019 | Greece |
| MH460600.1 / O19 | H57 | Tsaparis et al., 2019 | Greece |
| MH460601.1 / O20 | H42 | Tsaparis et al., 2019 | Greece |
| MH460602.1 / P01 | H94 | Tsaparis et al., 2019 | Greece |
| MH460603.1 / P02 | H93 | Tsaparis et al., 2019 | Greece |
| MH460604.1 / P03 | H94 | Tsaparis et al., 2019 | Greece |
| MH460605.1 / P04 | H93 | Tsaparis et al., 2019 | Greece |
| MH460606.1 / P05 | H94 | Tsaparis et al., 2019 | Greece |
| MH460607.1 / P06 | H94 | Tsaparis et al., 2019 | Greece |
| MH460608.1 / P07 | H94 | Tsaparis et al., 2019 | Greece |
| MH460609.1 / P08 | H94 | Tsaparis et al., 2019 | Greece |
| MH460610.1 / P09 | H94 | Tsaparis et al., 2019 | Greece |
| MH460611.1 / P10 | H94 | Tsaparis et al., 2019 | Greece |
| MH460612.1 / P11 | H94 | Tsaparis et al., 2019 | Greece |
| MH460613.1 / P12 | H94 | Tsaparis et al., 2019 | Greece |
| MH460614.1 / P13 | H94 | Tsaparis et al., 2019 | Greece |
| MH460615.1 / P14 | H94 | Tsaparis et al., 2019 | Greece |
| MH460616.1 / P15 | H94 | Tsaparis et al., 2019 | Greece |
| MH460617.1 / P16 | H94 | Tsaparis et al., 2019 | Greece |
| MH460618.1 / P17 | H94 | Tsaparis et al., 2019 | Greece |
| MH460619.1 / P18 | H94 | Tsaparis et al., 2019 | Greece |
| MH460620.1 / T01 | H145 | Tsaparis et al., 2019 | Greece |
| MH460621.1 / V01 | H146 | Tsaparis et al., 2019 | Greece |
| MH460622.1 / V02 | H147 | Tsaparis et al., 2019 | Greece |
| MH460623.1 / V03 | H146 | Tsaparis et al., 2019 | Greece |
| MH460624.1 / V05 | H146 | Tsaparis et al., 2019 | Greece |
| MH460625.1 / Z01 | H140 | Tsaparis et al., 2019 | Greece |
| MH460626.1 / Z02 | H95 | Tsaparis et al., 2019 | Greece |
| MH460627.1 / Z05 | H145 | Tsaparis et al., 2019 | Greece |
| MH460628.1 / Z07 | H145 | Tsaparis et al., 2019 | Greece |
| MH460629.1 / Z08 | H145 | Tsaparis et al., 2019 | Greece |
| MH460630.1 / Z09 | H95 | Tsaparis et al., 2019 | Greece |
| MH460631.1 / Z10 | H145 | Tsaparis et al., 2019 | Greece |
| MH460632.1 / Z11 | H145 | Tsaparis et al., 2019 | Greece |
| MH460633.1 / Z12 | H145 | Tsaparis et al., 2019 | Greece |
| MH460634.1 / Z13 | H145 | Tsaparis et al., 2019 | Greece |
| MH460635.1 / Z14 | H145 | Tsaparis et al., 2019 | Greece |
| MH460636.1 / Z15 | H95 | Tsaparis et al., 2019 | Greece |
| MH460637.1 / Z16 | H148 | Tsaparis et al., 2019 | Greece |
| MH460638.1 / Z17 | H95 | Tsaparis et al., 2019 | Greece |
| KY114497.1 / C1 | H63 | Stefanovic et al., 2019 | Serbia |
| KY114498.1 / C2 | H49 | Stefanovic et al., 2019 | Serbia |
| KY114499.1 / C3 | H38 | Stefanovic et al., 2019 | Serbia |
| KY114500.1 / C4 | H45 | Stefanovic et al., 2019 | Serbia |
| KY114501.1 / C5 | H40 | Stefanovic et al., 2019 | Serbia |
| KY114502.1 / C6 | H53 | Stefanovic et al., 2019 | Serbia |
| KY114503.1 / C7 | H54 | Stefanovic et al., 2019 | Serbia |
| KY114504.1 / C8 | H42 | Stefanovic et al., 2019 | Serbia |
| KY114505.1 / C9 | H38 | Stefanovic et al., 2019 | Serbia |
| KY114506.1 / C10 | H149 | Stefanovic et al., 2019 | Serbia |
| KY114507.1 / C11 | H150 | Stefanovic et al., 2019 | Serbia |
| KY114508.1 / C12 | H57 | Stefanovic et al., 2019 | Serbia |
| KY114509.1 / C13 | H54 | Stefanovic et al., 2019 | Serbia |
| KY114510.1 / C14 | H151 | Stefanovic et al., 2019 | Serbia |
| KY114511.1 / C15 | H29 | Stefanovic et al., 2019 | Serbia |
| KY114512.1 / C16 | H39 | Stefanovic et al., 2019 | Serbia |
| KP659198.1 / 006 | H16 | Nemeth et al., (unpub) | Hungary |
| KP659199.1 / 003 | H16 | Nemeth et al., (unpub) | Hungary |
| KP659200.1 / 029 | H16 | Nemeth et al., (unpub) | Hungary |
| KP659201.1 / 008 | H16 | Nemeth et al., (unpub) | Hungary |
| KP659202.1 / 024 | H16 | Nemeth et al., (unpub) | Hungary |
| KP659203.1 / 033 | H16 | Nemeth et al., (unpub) | Hungary |
| KP659204.1 / 173 | H16 | Nemeth et al., (unpub) | Hungary |
| KP659205.1 / 007 | H29 | Nemeth et al., (unpub) | Hungary |
| KP659206.1 / 171 | H29 | Nemeth et al., (unpub) | Hungary |
| KP659207.1 / 067 | H152 | Nemeth et al., (unpub) | Hungary |
| KP659208.1 / 005 | H16 | Nemeth et al., (unpub) | Hungary |
| KP659209.1 / 072 | H153 | Nemeth et al., (unpub) | Hungary |
| KP659210.1 / 073 | H153 | Nemeth et al., (unpub) | Hungary |
| KP659211.1 / 075 | H53 | Nemeth et al., (unpub) | Hungary |
| KP659212.1 / 088 | H53 | Nemeth et al., (unpub) | Hungary |
| KP659213.1 / 096 | H53 | Nemeth et al., (unpub) | Hungary |
| KP659214.1 / 066 | H40 | Nemeth et al., (unpub) | Hungary |
| KP659215.1 / 084 | H40 | Nemeth et al., (unpub) | Hungary |
| KP659216.1 / 014 | H40 | Nemeth et al., (unpub) | Hungary |
| KP659217.1 / 068 | H54 | Nemeth et al., (unpub) | Hungary |
| KP659218.1 / 170 | H154 | Nemeth et al., (unpub) | Hungary |

**Table S3** - Genetic distances among sampling locations within genetic Iberia, inferred from microsatellite data. Lower left matrix – distances estimated according to Slatkin (1995). Upper right matrix – distances estimated according to Nei (1978). Underlined values correspond to non-significant distances among sampling locations (p<0.05).

|  | **Alentejo** | **Beiras** | **Peneda Gerês** | **Trás-os-Montes** | **Valsemana** | **Astúrias** | **Galiza** |
| --- | --- | --- | --- | --- | --- | --- | --- |
| **Alentejo** |  | 0.094 | 0.511 | 0.380 | 0.413 | 0.354 | 0.330 |
| **Beiras** | 0.033 |  | 0.368 | 0.237 | 0.286 | 0.183 | 0.208 |
| **Minho** | 0.202 | 0.159 |  | 0.124 | 0.227 | 0.172 | 0.119 |
| **Trás-os-Montes** | 0.139 | 0.096 | 0.074 |  | 0.138 | 0.250 | 0.063 |
| **Valsemana** | 0.145 | 0.111 | 0.118 | 0.055 |  | 0.124 | 0.070 |
| **Astúrias** | 0.127 | 0.078 | 0.094 | 0.103 | 0.049 |  | 0.094 |
| **Galiza** | 0.114 | 0.079 | 0.070 | 0.024 | 0.034 | 0.032 |  |

**Table S4** – Evidences for genotyping bias caused by the presence of null alleles, allele dropout or stuttering, estimated using Micro-checker, for each of the populations defined *a posteriori*. *Null* stands for evidence of null alleles, *Stutter* for stuttering and *Drop* for allele dropout. Significant evidence is signaled in bold underlined **sig** and absence of significant evidence is signaled by 0.

|  | **CS Portugal (n=20)** | | | **Peneda Gerês NP (n=33)** | | | **Trás-os-Montes (n=14)** | | | **NW Iberia (n=22)** | | | **Valsemana (n=19)** | | |
| --- | --- | --- | --- | --- | --- | --- | --- | --- | --- | --- | --- | --- | --- | --- | --- |
| **Marker** | **Null** | **Stutter** | **Drop** | **Null** | **Stutter** | **Drop** | **Null** | **Stutter** | **Drop** | **Null** | **Stutter** | **Drop** | **Null** | **Stutter** | **Drop** |
| **NVHRT48** | 0 | 0 | 0 | 0 | 0 | 0 | 0 | 0 | 0 | 0 | 0 | 0 | 0 | 0 | 0 |
| **NVHRT16** | 0 | 0 | 0 | 0 | 0 | 0 | 0 | 0 | 0 | 0 | 0 | 0 | 0 | 0 | 0 |
| **CSSM66** | **sig** | 0 | 0 | 0 | 0 | 0 | 0 | 0 | 0 | 0 | 0 | 0 | **sig** | 0 | 0 |
| **INRA006** | 0 | 0 | 0 | 0 | 0 | 0 | 0 | 0 | 0 | 0 | 0 | 0 | 0 | 0 | 0 |
| **RT1** | **sig** | 0 | 0 | 0 | 0 | 0 | 0 | 0 | 0 | 0 | 0 | 0 | 0 | 0 | 0 |
| **BM6505** | 0 | 0 | 0 | 0 | 0 | 0 | 0 | 0 | 0 | 0 | 0 | 0 | 0 | 0 | 0 |
| **NVHRT21** | 0 | 0 | 0 | 0 | 0 | 0 | 0 | 0 | 0 | 0 | 0 | 0 | 0 | 0 | 0 |
| **BM1818** | 0 | 0 | 0 | 0 | 0 | 0 | **sig** | 0 | 0 | 0 | 0 | 0 | **sig** | 0 | 0 |
